# Using clinical parameters to guide fluid therapy in high-risk thoracic surgery. A retrospective, observational study

**DOI:** 10.1186/s12871-015-0072-2

**Published:** 2015-06-12

**Authors:** Lars Stryhn Bjerregaard, Hasse Møller-Sørensen, Kristoffer Lindskov Hansen, Jesper Ravn, Jens Christian Nilsson

**Affiliations:** 1 Section for Surgical Pathophysiology & Department of Cardiothoracic Anaesthesiology, Rigshospitalet, University of Copenhagen, Blegdamsvej 9, DK-2100 Copenhagen, Denmark; 2 Department of Cardiothoracic Anaesthesiology, Rigshospitalet, University of Copenhagen, Blegdamsvej 9, DK-2100 Copenhagen, Denmark; 3 Department of Radiology, Rigshospitalet, University of Copenhagen, Blegdamsvej 9, DK-2100 Copenhagen, Denmark; 4 Department of Cardiothoracic Surgery, Rigshospitalet, University of Copenhagen, Blegdamsvej 9, DK-2100 Copenhagen, Denmark; 5 Department of Cardiothoracic Anaesthesiology, Rigshospitalet, University of Copenhagen, Blegdamsvej 9, DK-2100 Copenhagen, Denmark

**Keywords:** Fluid therapy, Perioperative care, Pneumonectomy, Pulmonary edema, Postoperative complications

## Abstract

**Background:**

Despite extensive research, the debate continues as to the optimal way of guiding intraoperative and postoperative fluid therapy. In 2009 we changed our institutional guideline for perioperative fluid therapy in patients undergoing extrapleural pneumonectomy (EPP) and implemented the use of central venous oxygen saturation and intended low urine output to guide therapy in the early postoperative period. Here we evaluate the consequences of our changes.

**Methods:**

Retrospective, observational study of 30 consecutive patients undergoing EPP; 18 who had surgery before and 12 who had surgery after the changes. Data were collected from patient files and from institutional databases. Outcome measures included: Volumes of administered fluids, fluid balances, length of stays and postoperative complications. Dichotomous variables were compared with Fisher’s exact test, whereas continuous variables were compared with Student’s unpaired *t*-test or the Wilcoxon Two-Sample Test depending on the distribution of data.

**Results:**

The applied changes significantly reduced the volumes of administered fluids, both in the intraoperative (*p* = 0.01) and the postoperative period (*p* = 0.04), without increasing the incidence of postoperative complications. Mean length of stay in the intensive care unit (LOSI) was reduced from three to one day (*p* = 0.04) after the changes.

**Conclusion:**

The use of clinical parameters to balance fluid restriction and a sufficient circulation in patients undergoing EPP was associated with a reduction in mean LOSI without increasing the incidence of postoperative complications. Due to methodological limitations these results are only hypothesis generating.

## Background

With morbidity and mortality rates of 22–82 % and 3–34 % respectively [1, 2], extrapleural pneumonectomy (EPP) is considered a high-risk procedure. Acute Respiratory Distress Syndrome (ARDS) [3] is one of several well-known complications to EPP, and since excessive fluid administration may play a role in the development of ARDS [4], perioperative fluid administration tends to be restrictive in these procedures. However, the fluid and inopressor therapy has historically been quite variable in patients undergoing EPP, which were why we in August 2009 changed our institutional guideline for perioperative fluid therapy in these patients. The purpose was to optimise the perioperative treatment by balancing restrictive fluid administration against an adequate circulation to ensure sufficient oxygen supply. The changes included steps to restrict intraoperative fluid administration and the implementation of a clinically based algorithm of balanced goal-directed therapy (BGDT) to use postoperatively in the intensive care unit (ICU). The objective of this study was to assess the consequences of our changes in terms of volumes and balances of administered fluids, incidences of postoperative complications, length of stay in the ICU (LOSI) and length of stay in the hospital (LOS).

## Methods

### Ethics, study design, settings and participants

This study was considered a quality assurance process for which reason approval by the regional ethical committee was not required. Collection of data was approved by the Danish Data Protection Agency (J.nr: 2007-58-0015).

This is a retrospective, observational study, including 30 consecutive patients who underwent EPP for malignant pleural mesothelioma between August 2007 and January 2012 at the Copenhagen University Hospital, Rigshospitalet, Denmark. The changed guideline was introduced in August 2009, and 18 patients underwent surgery before (before group), while 12 patients underwent surgery after the changes were implemented (after group). Before surgery, all patients received 3–6 series of pemtrexed and cisplatin chemotherapy and afterwards operability was confirmed by a positron emission tomography/computed tomography scan. A mediastinoscopy was performed one week before EPP was scheduled, and EPP was offered in case of: unilateral cancer without chest wall penetration or infiltrated lymph nodes in the mediastinum, epithelial type of cancer, pneumonectomy allowed by pulmonary function, no major cardiac events in the past, a left ventricular ejection fraction > 40 % and age < 70 years.

The patients were anaesthetised using propofol and remifentanil, and all patients had a left-sided, double-lumen endotracheal tube, an arterial line and a central venous line inserted. During one-lung ventilation, pressure-controlled ventilation was used with the fraction of oxygen reduced to the lowest level keeping arterial saturation above 90 %. All patients were extubated in the operating room and transferred to the ICU, and postoperative analgesia was achieved by a thoracic epidural catheter. The surgery was performed by the same experienced surgeon through a posterolateral thoracotomy and the same ICU discharge criteria were utilised during the study period [5].

### Changes in standard therapy

A number of changes were made to balance restrictive fluid administration and adequate oxygen supply. Fluid restriction during surgery was achieved by simple procedural changes, as outlined below, whereas postoperative fluid restriction was reached through the introduction of BGDT.

Changes applied in the intraoperative period:

1. Hypotension related to the induction of anaesthesia or to epidural analgesia was treated with ephedrine and/or phenylephrine rather than intravenous fluid administration.

2. Insensible perspiration was reduced from 5 ml * kg^−1^ * hour (h)^−1^ to 1 ml * kg^−1^ * h^−1^, and volumes of intravenously administered medicine was included in the calculation of the intraoperative fluid balance, which were intended between 0 and minus 500 ml.

**Fig. 1:**
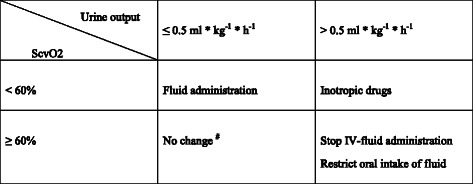
Schematic model of balanced goal-directed therapy used in the postoperative period after extrapleural pneumonectomy. ^#^In the case of total anuria in combination with ScvO2 ≥ 60 % kidney failure should be suspected. *ScvO2* Central venous oxygen saturation; *ml* millilitres; *kg* kilogram (ideal body weight); *h* hour; *IV* intravenous

Change applied in the postoperative period:

1. BGDT was implemented to achieve fluid restriction and still maintain adequate oxygen delivery. The intention was to guide therapy by easily measured clinical variables. The interventional thresholds were urine output of 0.5 ml * kg^−1^ * h^−1^ and central venous oxygen saturation (ScvO2) of 60 % (Fig. 1).

Changes applied in the intra- and postoperative periods:

1. The recommended colloid of choice was changed from dextran (Macrodex®, MEDA, Solna Sweden) to hydroxyethyl starch (Voluven®, Fresenius Kabi AG, Bad Homburg, Germany) as the maximal volume of Voluven® to be administered in 24 h is twice that of Macrodex®.

2. It was pointed out to all relevant personnel that the transfusion trigger for red blood cells is a haemoglobin concentration of 7.5 g * dl^−1^ in the case of no bleeding or controlled bleeding [6].

### Data collection

Data on age, gender, weight, height, smoking status, comorbidity, classification according to the American Society of Anesthesiologists (ASA), plasma creatinine preoperatively and on the first and third postoperative day (POD), values of ScvO2, plasma lactate, administration of inotropic drugs and cumulated urine output on the day of surgery, administered volumes of fluid (including blood products), and fluid balances were collected from the patients’ files. In all cases with LOSI > 1 day, we reviewed the patient’s medical chart to determine the main reason for the continued stay in the ICU. Preoperative pulmonary status (forced expiratory volume in one second (FEV1) and diffusion capacity for carbon monoxide (DLCO)), tumour stage, resection side, duration of surgery, intraoperative bleeding, LOSI, LOS and postoperative complications were collected from institutional databases. Measurements of ScvO2 and plasma lactate levels were obtained regularly. Chest x-rays from the first POD were evaluated by a radiologist for signs of pulmonary stasis. Fluid balances were calculated as the sum of all fluids given, subtracted all deficits such as perspiration, urine output, output in drain(s) and other losses if relevant. If a patient was readmitted to the ICU < 24 h after discharge, it was considered a continued admission. LOSI and LOS were both counted from the day of surgery. Postoperative complications were defined as complications before discharge from the hospital.

### Statistics

Dichotomous variables are given as counts and percentages. Continuous variables are given as means with 95 % confidence intervals or as medians and ranges in case of non-normally distributed data. Dichotomous variables were compared with Fisher’s exact test, whereas continuous variables were compared with Student’s unpaired *t*-test or the Wilcoxon Two-Sample Test depending on the distribution of data. All tests were 2-sided, and a significance level of 5 % was used. Data analyses were performed in the Statistical Analysis System version 9.1.3 (SAS Institute Inc., Cary, NC).

## Results

The two groups were comparable regarding basic characteristics (Table 1). Volumes of administered fluids and fluid balances are given in Table 2, and data from the intra- and postoperative periods are given in Table 3. During surgery, 14 patients (78 %) in the before group and five patients (42 %) in the after group had blood transfusions (*p* = 0.06). Postoperatively in the ICU eight patients (44 %) in the before group and one patient (8 %) in the after group received blood products (*p* = 0.05). We found no differences in the incidence of cardiac, respiratory, gastrointestinal or renal complications between the two groups (Table 3). Patients in the after group had significantly shorter LOSI (*p* = 0.04), and a tendency towards shorter LOS (*p* = 0.09) (Table 3). Six patients from the before group had LOSI > 1 day; five due to a continued need for inotropic support and one who developed postoperative atrial fibrillation. None of the patients in the after group had LOSI > 1 day (Table 3). One patient from the before group was discharged from the ICU on the second postoperative day, but readmitted within 24 h. This patient needed ventilator treatment for 19 days. Also, one patient from the before group required continuous renal replacement therapy for 24 h.

**Table 1:** Basic characteristics

	Before group (n = 18)		After group (n = 12)		*p* value
Age (years) ^a^	62 (47; 68)		58 (43; 67)		0.19
Gender ^c^					1.00
Female	5 (28)		3 (25)		
Male	13 (72)		9 (75)		
Weight (kg) ^b^	82 (74; 89)		83 (72; 94)		0.83
Height (cm) ^b^	176 (172; 180)		178 (172; 184)		0.48
BMI > 30 ^c^	2 (11)		2 (17)		1.0
Smoking status ^c^					1.0
Current	4 (22)		2 (17)		
Former	7 (39)		5 (41)		
Never	6 (33)		5 (42)		
Data missing	1 (6)		0		
ASA-classification ^c^					1.0
ASA I	1 (6)		0		
ASA II	12 (67)		8 (67)		
ASA III	5 (28)		4 (33)		
Comorbidity ^c^					
COPD	0		0		
Hypertension	4 (22)		3 (25)		
IHD	1 (6)		0		
DM	1 (6)		2 (17)		
Resection side^c^					0.71
Right	8 (44)		7 (58)		
Left	10 (56)		5 (42)		
TNM-classification^c^	Pre-operative	Postoperative	Pre-operative	Postoperative	
T1N0M0	2 (6)	0	0	0	
T2N0M0	12 (67)	3 (17)	9 (75)	1 (8)	
T3N0M0	4 (22)	12 (67)	1 (8)	3 (25)	
T3N1M0	0	0	0	2 (17)	
T3N2M0	0	1 (6)	1 (8)	5 (42)	
T4N0M0	0	1 (6)	1 (8)	1 (8)	
T4N1M0	0	1 (6)	0	0	
Preoperative FEV1^b^ (% of expected)	87 [80; 94]		77 [68; 85]		0.07
Preoperative DLCO^b^ (% of expected)	71 [66; 76]		71 [63; 78]		0.93

*ASA* American Society of Anesthesiologists; *BMI* Body Mass Index; *cm* centimetres; *COPD* Chronic Obstructive Pulmonary Disease; *DLCO* Diffusing Capacity for Carbon Monoxide; *DM* Diabetes Mellitus; *FEV1* Forced Expiratory Volume in one second; *IHD* Ischemic Heart Disease; *kg* kilograms; *TNM* Tumour, Node, Metastasis International Staging System for Lung Cancer

^a^Median (range), ^b^Mean (95 % confidence interval), ^c^Number of patients (group percentage)

**Table 2:** Volumes of administered fluids and fluid balances during and after extrapleural pneumonectomy^a^

	Intra-operative			ICU on the day of operation		
	Before group (n = 18)	After group (n = 12)	*P* value	Before group (n = 18)	After group (n = 12)	*P* value
Red blood cells^b^	300 (0; 1200)	0 (0; 600)		0 (0; 600)	0 (0; 300)	
Fresh frozen plasma^b^	0 (0; 600)	0 (0; 600)		0 (0; 600)	None	
Colloids	950 (500; 1750)	500 (0; 1500)		550 (0; 1500)	500 (0; 1500)	
Crystalloids	1150 (500; 2500)	1000 (500; 1500)		1713 (625; 3075)	1513 (325; 1975)	
Total volume of administered fluid	2675 (1000; 5350)	1600 (600; 3600)	0.01	2588 (1400; 4925)	1810 (825; 3525)	0.04
Fluid balances^c^	779 (−429; 1863)	−385 (−2130; 10)	0.0003	176 (−970; 1567)	111 (−419; 287)	0.26

All values are given in ml, as median (range). Colloids are dextran, hydroxyethyl starch or 5 % human albumin. Crystalloids are isotonic solutions of crystalloids with or without glucose

*ICU* Intensive Care Unit; *ml* millilitres

^a^Thirty patients divided in two groups according to whether they had surgery before or after changes in standard therapy, ^b^One unit = 300 ml, ^c^The calculations in both groups included insensible perspiration of 1 ml/kg/hour

**Table 3:** Data from the intra- and postoperative period in thirty patients undergoing extrapleural pneumonectomy (EPP)

	Before group (n = 18)		After group (n = 12)		*P* value
Duration of surgery (minutes)^a^	220 (185; 315)		239 (162; 305)		0.17
Bleeding (millilitres)^a^	1000 (600; 2400)		1065 (250; 1600)		0.49
Cumulated urine output on the day of surgery^a^	1810 (1240; 2995)		1528 (945; 2320)		0.01
Values of ScvO2, obtained in the ICU on the day of surgery^a, b^					
Lowest values in the period	61.8 % (42.7; 71.2)		60.2 % (56.6; 74.1)		0.45
Highest values in the period	65.0 % (60.1; 77.0)		70.6 % (62.5; 78.6)		0.05
Needed inopressor therapy in the ICU^c^	8 (44) ^d^		3 (25)^e^		0.44
Pulmonary stasis on X-ray POD 1^c^	1 (6)		3 (25)		0.27
Postoperative complications^c^					
Arial Fibrillation	10 (56)		5 (42)		0.71
Plasma Lactate > 2.5 mmol/l^f^	5 (28)		3 (25)		1.0
Pneumonia	2 (11)		0		0.50
Respiratory insufficiency	1 (6)		0		1.0
Gastrointestinal complications^g^	2 (11)		3 (25)		0.36
Serum Creatinine	POD 1	POD 3	POD 1	POD 3	
Increased 1.5–2 x preoperative level	0	3 (17)	1 (8)	1 (8)	0.40/0.63
Increased 2–3 x preoperative level	1 (6)	1 (6)	1 (8)	1 (8)	1.00/1.00
Increased more than 3 x preoperative level	0	1 (6)	0	0	--/1.0
Needed haemodialysis or haemofiltration	1 (6)		0		1.0
Readmissions to the ICU^f^	1 (6)		0		1.0
Thirty days mortality^f^	1 (6)		0		1.0
Length of stay in the ICU (days)^a^	1 (1; 25)		1 (1; 1)		0.04
Length of stay in hospital (days)^a^	16.5 (8; 42)		13 (10; 24)		0.09

*ICU* Intensive Care Unit; *POD* Postoperative Day; *ScvO2* Central venous saturation

^a^Median (range), ^b^n = 16 in the before group and n = 11 in the after group, ^c^ Number of patients (group percentage), ^d^All 8 patients received continuous infusion of dopamine between six and 72 h, and one patient needed supplementary infusion of norepinephrine for six hours, ^e^All three patients received continuous infusion of dopamine between 10 and 11 h, ^f^ Values obtained on the day of operation, ^g^Pseudo obstruction or severe constipation

## Discussion

The development of ARDS as characterised in the Berlin Definition from 2011 [3] is a well-known complication to pulmonary resection, and to pneumonectomy in particular. In 1984, Zeldin [7] proposed that excessive fluid administration was the cause of pulmonary oedema after pneumonectomy. Since then several mechanisms have been proposed to take part in the development of what may be defined as ARDS after pneumonectomy, and excessive fluid administration might just be one of several contributory factors [4].

In 2002, Møller et al. [8] showed that an excess fluid balance of more than 4 l during surgery was associated with a higher risk of postoperative complications after pneumonectomy and in 2010, Marret et al. [9] found that liberal fluid administration during surgery was a risk factor for major complications after pneumonectomy. Furthermore, Suehiro et al. [10] found that high fluid infusion volumes during pneumonectomy were a risk factor for developing postoperative right-sided heart failure. However, even though fluid restriction seems to be an important factor in reducing complications after pneumonectomy, it may compromise cardiac output and ultimately lead to inadequate oxygen delivery. As recently discussed [11], the use of haemodynamic monitoring by devices such as transesophageal Doppler, PiCCO etc. may prove useful for guiding perioperative fluid therapy in pulmonary resection surgery, but still further research is needed in this field. However, goal-directed therapy using measurements of cardiac output alone, often leads to higher volumes of administered fluid compared to conventional therapy [12], and therefore the debate continues as to the optimal dose of fluid and which haemodynamic goal and/or vasopressor are best [13].

According to the Fick equation mixed venous oxygen saturation (SvO2) is related to the fraction of delivered oxygen that is consumed by the tissues, and a reduced SvO2 is thought to reflect elevated oxygen extraction, compensating for a reduced cardiac output [14]. Since ScvO2 has been shown to correlate with SvO2 [15], the former was used to avoid insertion of a pulmonary artery catheter. In sepsis treatment, a ScvO2 > 70 % is widely used and it has been suggested that normovolemia in the supine position is reached when fluid administration results in a ScvO2 higher than 65 % [16]. Furthermore, it has been suggested that perioperative mean values of ScvO2 < 71–73 % are correlated to an increased risk of postoperative complications - predominantly infections - after high-risk abdominal surgery [17, 18]. Consequently, a ScvO2 of 60 % as interventional threshold could be considered to be a rather low. However, in pulmonary resection surgery, the main concern is still the development of acute lung injury and ARDS [11], which is why the main purpose of implementing BGDT was to avoid hypervolemia by restricting fluid administration without compromising the circulation and oxygen delivery. Thus, due to the possible hazardous consequences of hypervolemia in patients undergoing EPP a relatively low interventional set point for ScvO2 was chosen. Also, it has been suggested that the optimal interventional threshold values of ScvO2 are dependent on the study population [17] which is why future prospective studies are needed to establish optimal target values of ScvO2 adapted to specific surgical populations.

Attempting to minimize the total expansion of body interstitial fluids, we wished to restrict the use of crystalloids but at the same time avoiding the use of blood products for volume substitution. Therefore, we changed the recommended colloid of choice from dextran to hydroxyethyl starch, allowing administration of a higher dose in 24 h. We acknowledge that the use of synthetic colloids is controversial due to results from multicentre studies on sepsis treatment [19, 20], but similar studies in surgical patients are still anticipated. Beside the change of colloid, the new guidelines also clearly emphasised the restrictive use of blood products which may have contributed to the observed tendency towards reduced volumes of administered blood products and the reduced proportion of patients, who received blood transfusion in the after group. Also, the total volume of intraoperative fluids were reduced (Table 3) resulting in less hemodilution.

Radiological signs of pulmonary stasis were present on chest X-rays from the first POD in one patient from the before group and in three patients from the after group, but none of the four patients had clinical symptoms of pulmonary stasis or oedema, and all were discharged from the ICU on POD 1. The implementation of BGDT did not seem to increase the incidence of kidney injury according to the creatinine criteria of the RIFLE-classification [21] neither did it increase the use of inotropic agents in the ICU (Table 3).

A limitation to this retrospective, observational study is that the impact of the changes to standard therapy, were compared to a group of historical, non-matched patients and therefore the results could be dependent on non-measured variables among patients. However, this group of patients is a highly selected group without significant comorbidity and the data were comparable regarding basic characteristics, suggesting an acceptable matching between groups. Furthermore, the main part of the data was collected from electronic databases, which might result in a higher level of data validity and completeness. Another limitation is that several changes were applied at the same time, which prevents us from drawing conclusions on the impact of the individual changes. Also, our results could be influenced by variables of clinical, technical, or logistic origin that were not controlled during the study period. Finally, the small sample size and hereby potential lack of statistically power precludes us from drawing conclusions on risk and benefits and therefore our results should be considered hypothesis generating, serving for designing further investigations.

## Conclusion

In this retrospective study, a number of perioperative steps to limit fluid administration in patients undergoing EPP without compromising circulation and oxygen supply were associated with a reduction in mean LOSI without increasing the incidence of postoperative complications. Our results suggests that it may be safe to use simple clinical parameters in guiding fluid therapy in high-risk surgery, but due to methodological limitations these results are only hypothesis generating.

## Abbreviations

ASA: American Society of Anesthesiologists
ARDS: Acute respiratory distress syndrome
BGDT: Balanced goal-directed therapy
EPP: Extrapleural pneumonectomy
FEV1: Forced expiratory volume in one second
DLCO: Diffusion capacity for carbon monoxide
ICU: Intensive care unit
LOS: Length of stay in the hospital
LOSI: Length of stay in the ICU
POD: Postoperative ay
SvO2: Mixed venous oxygen saturation
ScvO2: Central venous oxygen saturation
